# Pneumothorax in newborns: a cohort study from three NICUs

**DOI:** 10.1038/s41372-025-02408-9

**Published:** 2025-09-04

**Authors:** Mohsen A. A. Farghaly, Mahmoud A. M. Ali, Ceyda Acun, Vanishree Nandakumar, Hatem Eltaly, Mohamed A. Mohamed, Hany Aly

**Affiliations:** 1 Neonatology Division, Cleveland Clinic Children’s, Cleveland, OH USA; 2 West Virginia University School of Medicine, Morgantown, WV USA

**Keywords:** Respiratory tract diseases, Risk factors

## Abstract

**Objective:**

To assess pneumothorax prevalence in neonates across gestational age (GA) categories, identify associated variables, and examine the impact of bubble CPAP (b-CPAP) implementation.

**Study design:**

A cohort of 58,706 infants born at three hospitals over six years was analyzed, grouped by GA: ≥35 weeks, 29–34 weeks, and ≤28 weeks. Pneumothorax cases were matched with controls, and prevalence before and after b-CPAP adoption was compared.

**Results:**

Pneumothorax occurred in 310 infants (0.53%): 0.39% in ≥35 weeks, 4.0% in 29–34 weeks, and 4.6% in ≤28 weeks GA. Most cases occurred within 24 hours of birth, especially in ≥35 weeks (76%). In the ≥35-week group, pneumothorax was associated with male sex, chorioamnionitis, and delivery room CPAP. In the 29–34-week group, it was linked to small for gestational age, maternal diabetes, and surfactant use. In ≤28-week infants, delivery room intubation was the primary risk factor. Pneumothorax prevalence in non-intubated infants was unchanged after b-CPAP implementation (0.35% vs. 0.41%; aOR = 0.85, 95% CI: 0.62–1.16; *P* = 0.35).

**Conclusion:**

Delivery room interventions, not NICU b-CPAP use, were associated with neonatal pneumothorax.

## Introduction

Pneumothorax is a serious condition with potentially severe consequences depending on the timing, severity and clinical setting, and frequently seen in the neonatal intensive care unit (NICU) [1]. The incidence of pneumothorax varies according to gestational age, baseline respiratory disease, and the level of respiratory support such as the use of positive pressure ventilation (PPV), mechanical ventilation, or continuous positive airway pressure (CPAP) [2, 3]. The incidence of pneumothorax among NICU admissions ranges from 0.5% to 2% and is associated with an increased risk of mortality; however, the exact magnitude of this risk remains unclear due to limited published data [4]. Maneuvers to closely monitor tidal volume (TV) and peak inspiratory pressure (PIP) during invasive mechanical ventilation was shown to be efficacious in decreasing the incidence of pneumothorax in preterm infants [5]. However, despite advances in neonatal respiratory care, pneumothorax remains a significant concern due to its impact on neonatal morbidity and mortality [6].

Although there are multiple causes for respiratory distress in neonates, two broad categories can be identified: transient respiratory illness that usually occurs in late preterm and term infants, and alveolar prematurity/surfactant deficiency that is mainly observed in premature infants. Bubble CPAP (b-CPAP) has been used for decades for respiratory support in premature infants [7]. However, the efficacy and complications in late preterm and term infants are rarely studied. A recent cohort study suggested an increased incidence of pneumothorax in term and late preterm infants resuscitated with CPAP in the delivery room [8]. However, the association between pneumothorax and respiratory management beyond delivery room resuscitation, including subsequent respiratory support in the NICU such as b-CPAP, has not been investigated.

The relationship between pneumothorax and the type of respiratory support in late preterm infants is lacking. Differences in CPAP devices and strategies in the NICU, such as b-CPAP necessitate further investigation regarding their association with pneumothorax. Understanding the risk factors, timing and clinical course of pneumothorax in preterm and term infants is essential for optimizing prevention and intervention strategies like the need for chest tube placement.

We hypothesized that the development of pneumothorax is more determined by the baseline respiratory illness and the respiratory support in both preterm and term infants distinctly. Thus, this study aimed to evaluate the prevalence of pneumothorax in neonates at different gestational age (GA) categories (≥ 35 0/7 weeks, 29 0/7-34 6/7 weeks, and ≤ 28 6/7 weeks), identify the risk factors associated with pneumothorax in the three GA categories, and examine whether b-CPAP adoption in the NICU is associated with pneumothorax. By systematically investigating these questions, we aimed to provide insights that could guide prevention/anticipation of neonatal pneumothorax.

## Methods

The study included a retrospective cohort of all infants born between January 1, 2017, and February 29, 2024, across three NICUs within the Cleveland Clinic Foundation, which collectively care for approximately 10,000 infants annually. This inclusive, population-based design was selected to ensure sufficient sample size and statistical power to detect clinically meaningful differences in pneumothorax prevalence across GA groups and to assess changes associated with the adoption of b-CPAP. Given the large underlying population, the cohort was expected to provide adequate power to detect modest effect sizes with narrow confidence intervals, particularly for rare outcomes such as pneumothorax. A formal power calculation was not performed prospectively, as all eligible infants during the study period were included to maximize statistical robustness. We reviewed the electronic medical records of the enrolled newborns. All data were collected via a standardized form and were entered into a secured REDCap program created specifically for this study. Data items were coded and de-identified before analysis.

We extracted the maternal and neonatal demographic and clinical characteristics from medical records. The study included all newborn infants of all GA who had pneumothorax diagnosis. All cases were reviewed, and the diagnosis was reassured via a chest radiograph accompanied by a radiologist report. We excluded patients with pneumothorax due to congenital anomalies of the lungs or airways, cardiothoracic surgical procedure, and/or congenital lobar emphysema. Controls without pneumothorax matched for GA and admission year were included for comparisons.

Clinical and demographic data were collected, and that included sex, race/ethnicity, birth weight (BW), method of delivery (C-section or vaginal), use of antenatal steroids, presence or absence of chorioamnionitis, maternal diabetes mellitus (DM), neonatal respiratory diseases, need of delivery room resuscitation, and need for respiratory support (oxygen, positive pressure ventilation (PPV), CPAP, administration of surfactant, highest and lowest peak inspiratory pressure (PIP), highest and lowest positive end expiratory pressure (PEEP), and endotracheal intubation.

### Bubble CPAP implementation

A structured implementation of bubble CPAP (b-CPAP) began in 2018. The program was first introduced at Hospital A in February 2018, initially targeting infants with a GA > 32 weeks and/or birth weight >1500 g. The implementation expanded in a stepwise fashion to include infants with younger GA, and by December 2018, b-CPAP was fully adopted in Hospital A. In January 2019, a similar implementation approach was initiated in Hospital B and completed by December 2019. By mid-2020, all three hospitals within the system had adopted b-CPAP. Prior to February 2018, infants were managed using various modes of non-invasive respiratory support, including ventilator-generated CPAP, infant flow systems, and biphasic CPAP, delivered via different facial interfaces such as nasal prongs, face masks, and RAM cannulas. After b-CPAP implementation, infants were exclusively supported with b-CPAP at a pressure of 5–6 cm H₂O, delivered via short, curved nasal prongs (Hudson RCI nasal prongs, Teleflex, Auckland, New Zealand; or Babi.Plus nasal prongs, Respiralogics, Reno, NV). The timing of b-CPAP implementation by unit and GA category was recorded, and the study population was stratified into two groups based on whether b-CPAP had been implemented. The incidence of pneumothorax was then compared between the pre- and post-implementation groups.

### Statistical analysis

We expressed categorical variables in numbers and percentages. Continuous parametric variables were expressed in mean and standard deviation and non-parametric variables in median and interquartile range. We divided the enrolled subjects into three groups based on gestational age, infants born ≥ 35 0/7 weeks, 29 0/7-34 6/7 weeks, and ≤ 28 6/7 weeks of gestation. We compared cases and controls and used logistic regression to control for the confounding variables that were significant in bivariate analyses. We further identified infants who were not supported by invasive mechanical ventilation at the time of pneumothorax and compared the prevalence of pneumothorax before and after b-CPAP implementation.

## Results

**Fig. 1:**
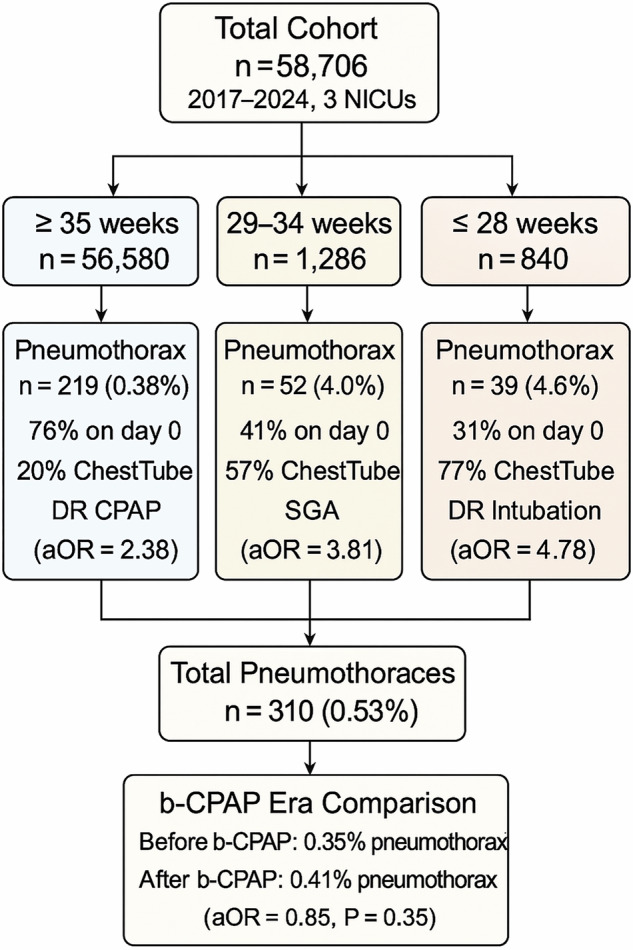
Flow chart for the study population (n = 58,706). Incidence, timing, chest tube placement, and key risk factors are shown. Independent associations included delivery room (DR) CPAP in infants ≥35 weeks (aOR = 2.38), small for gestational age (SGA) in 29–34 weeks (aOR = 3.81), and DR intubation in ≤28 weeks (aOR = 4.78). The overall pneumothorax rate was 0.53%. No significant difference was observed before vs. after bubble-CPAP (b-CPAP) implementation (0.35% vs. 0.41%; aOR = 0.85, *P* = 0.35).

A total of 58,706 were born during the study duration; of them 56,580 had GA ≥ 35 0/7 weeks, 1,286 had GA of 29 0/7-34 6/7 weeks, and 840 had GA ≤ 28 6/7 weeks. A total of 310 (0.53%) infants had pneumothorax. The prevalence of pneumothorax in the three GA categories was 0.39%, 4.0%, and 4.6% respectively (Fig. 1).

For newborns ≥ 35 weeks GA, most pneumothoraces (76%) occurred in the first 24 hours of life. Compared to controls, infants with pneumothorax had significantly less females [OR = 0.62 (CI:0.40-0.96) P = 0.03], as well as less late preterm infants (GA 35 0/7 - 36 6/7 weeks) [OR = 0.42 (CI:0.23-0.79) P = 0.008]. Pneumothorax was associated with maternal chorioamnionitis [OR = 2.07 (CI:1.13-3.79) P = 0.02], maternal antenatal steroids [OR = 5.22 (CI:2.09-13.0) P < 0.001], and delivery room CPAP [OR = 2.38 (CI:1.06-5.36) P = 0.04]. Among infants who required mechanical ventilation in the NICU, those who developed pneumothorax were exposed to higher PIP (20.6 ± 5 vs 14.3 ± 3 cmH2O, P = 0.005). Demographic and clinical characteristics of cases vs controls in the infants ≥ 35 weeks category are presented in Table 1.

**Table 1:** Demographic and clinical characteristics of infants ≥ 35 0/7 weeks.

	Cases n = 219	Controls n = 235	aOR, 95% CI, *p* value
Female sex	29.7	41.7	0.62 (0.40-0.96), 0.03
Caucasians race	59.8	54.9	1.24 (0.81-1.90), 0.33
Gestational age^a^	38.8 (1.6)	38.4 (1.7)	0.01
Gestational age <37 weeks	13.7	19.2	0.42 (0.23-0.79),0.008
Birth weight^a^	3208 (514)	3175 (558)	0.51
Birth weight <2500 g	8.22	10.2	0.64 (0.28-1.46),0.28
Small for GA status	10.5	8.51	2.10 (0.97-4.56), 0.06
Cesarean delivery	51.6	36.6	1.37 (0.89-2.10), 0.16
Maternal diabetes	19.2	15.7	1.22 (0.69-2.13), 0.49
Maternal chorioamnionitis	21.0	9.79	2.07 (1.13-3.79), 0.02
Maternal steroids	12.3	4.68	5.22 (2.09-13.0), <0.001
Meconium aspiration	20.6	14.9	0.81 (0.46-1.46), 0.49
Apgar score at 1 min^b^	8 (5,8)	8 (7,9)	<0.001
Apgar score at 5 min^b^	8 (7,9)	9 (8,9)	0.004
Delivery room oxygen	77.6	42.6	2.27 (0.99-5.14), 0.05
Delivery room CPAP	73.9	39.2	2.38 (1.06-5.36), 0.04
Delivery room positive ventilation	22.8	11.1	1.30 (0.70-2.44), 0.41
Delivery room intubation	5.94	3.83	1.19 (0.37-3.85), 0.77
Surfactant therapy	7.31	3.83	1.37 (0.51-3.66), 0.53
Average highest FiO_2_^a^	0.46 (.26)	0.48 (.30)	0.52
Average lowest FiO_2_^a^	0.27 (.10)	0.23 (.9)	0.004
Average highest PEEP^a^	5.2 (0.8)	5.4 (0.5)	0.41
Average lowest PEEP^a^	5.0 (0.5)	5 (0)	0.37
Average highest PIP^a^	20.6 (5)	14.3 (3)	0.005
Average lowest PIP^a^	17.8 (4.7)	12 (0.6)	<0.001

All values are presented as percentages except ^a^in mean (standard deviation) and ^b^in median (25,75 quartiles). *CPAP* continuous positive airway pressure, *FiO2* fraction of inspired oxygen, *PEEP* positive end expiratory pressure, *PIP* peak inspiratory pressure, *aOR* adjust odds ratios, and *CI* confidence intervals, that were calculated in a regression model.

For newborns 29-34 weeks GA, 41% of pneumothorax occurred in the first 24 hours of life, and 24% occurred in the second day of life. Compared to controls, pneumothorax was associated with small for gestational age (SGA) [OR = 3.81 (CI:1.30-11.2) P = 0.02], maternal diabetes [OR = 3.01 (CI:1.06-8.59) P = 0.04], and surfactant administration [OR = 6.43 (CI:2.16- 19.1) P = 0.001]. In the NICU, infants who developed pneumothorax had a prior exposure to higher FiO2 (P = 0.02) and higher PIP in those who were mechanically ventilated (19.8 ± 4.7 vs 13.4 ± 3.2 cmH2O, P = 0.003). Demographic and clinical characteristics of cases vs controls in the infants 29-34 weeks category are presented in Table 2.

**Table 2:** Demographic and clinical characteristics of infants 29 0/7 – 34 6/7 weeks.

	Cases n = 52	Controls n = 94	aOR, 95% CI, *p* value
Female sex	34.6	44.7	0.91 (0.36-2.30), 0.85
Caucasians race	65.4	50.0	2.30 (0.93-5.70), 0.07
Gestational age^a^	32.1 (1.8)	32.6 (1.7)	0.14
Birth weight^a^	1820 (427)	1921 (471)	0.20
Small for GA status	30.8	13.8	3.81 (1.30-11.2), 0.02
Cesarean delivery	71.2	69.2	0.66 (0.24-1.84), 0.43
Maternal diabetes	30.1	17.0	3.01 (1.06-8.59), 0.04
Maternal chorioamnionitis	9.62	7.45	1.43 (0.28-7.35), 0.67
Maternal steroids	67.3	77.7	0.32 (0.11-0.98), 0.04
Apgar score at 1 min^b^	7 (5,8)	7 (5,8)	0.97
Apgar score at 5 min^b^	8 (7,9)	8 (7,9)	0.35
Delivery room oxygen	98.1	65.9	35.0 (3.00-409), 0.005
Delivery room CPAP	86.5	69.2	0.85 (0.15-4.73), 0.85
DR room positive pressure ventilation	34.6	29.8	0.29 (0.09-0.89), 0.03
Delivery room intubation	7.69	4.26	0.58 (0.06-5.76), 0.64
Surfactant therapy	44.2	11.7	6.43 (2.16-19.1) 0.001
Average highest FiO2^a^	0.54 (30)	0.55 (31)	0.87
Average lowest FiO2^a^	0.31 (.18)	0.25 (.06)	0.02
Average highest PEEP^a^	5.7 (0.9)	5.1 (0.4)	0.01
Average lowest PEEP^a^	5.2 (0.7)	5.0 (0.0)	0.13
Average highest PIP^a^	19.8 (4.7)	13.4 (3.2)	0.003
Average lowest PIP^a^	18.5 (4.9)	12.9 (3.2)	0.01

All values are presented as percentages except ^a^in mean (standard deviation) and ^b^in median (25,75 quartiles). *CPAP* continuous positive airway pressure, *FiO2* fraction of inspired oxygen, *PEEP* positive end expiratory pressure, *PIP* peak inspiratory pressure, *aOR* adjust odds ratios, and *CI* confidence intervals, that were calculated in a regression model.

For newborns ≤ 28 weeks GA, 31% of pneumothorax occurred in the first day of life.

Mean age at diagnosis of pneumothorax was 3.1 ( ± 3) days. Compared to controls, pneumothorax was associated with delivery room intubation [OR = 4.78 (CI:1.02-22.4) P = 0.04], and higher PIP in the NICU (19.3 ± 5.6 vs 14.5 ± 34.2 cmH_2_O, P = 0.001). Demographic and clinical characteristics of cases vs controls in the infants ≤ 28 weeks category are presented in Table 3.

**Table 3:** Demographic and clinical characteristics of infants ≤ 28 6/7 weeks.

	Cases n = 39	Controls n = 44	aOR, 95% CI, *p* value
Female sex	46.2	52.3	0.65 (0.24-1.77), 0.40
Caucasians race	30.8	31.8	0.96 (0.32-2.89), 0.95
Gestational age^a^	25.9 (1.6)	25.9 (1.7)	0.90
Birth Weight^a^	817 (246)	789 (113)	0.59
Small for GA status	25.6	18.2	1.27 (0.39-4.14), 0.69
Cesarean delivery	82.1	68.2	2.69 (0.83-8.73), 0.10
Maternal diabetes	7.69	2.27	6.49 (0.47-91.6), 0.16
Maternal chorioamnionitis	23.1	27.3	0.52 (0.16-1.69), 0.28
Maternal steroids	89.7	79.6	3.51 (0.69-17.8), 0.28
Apgar score at 1 min^b^	2 (1,5)	3.5 (2,6.5)	0.09
Apgar score at 5 min^b^	6 (3,7)	7 (5,8)	0.06
Delivery room oxygen	100	97.7	0.99
Delivery room CPAP	61.5	56.8	2.70 (0.74-9.83), 0.13
DR room positive pressure ventilation	79.5	81.8	0.66 (0.12-3.57) 0.63
Delivery room intubation	66.7	52.3	4.78 (1.02-22.4), 0.04
Surfactant therapy	74.4	77.3	0.57 (0.14-2.24), 0.42
Postnatal steroids	38.5	36.4	0.95 (0.31-2.85), 0.92
Average highest FiO_2_^a^	0.84 (.25)	0.81 (.27)	0.59
Average lowest FiO_2_^a^	0.40 (.22)	0.29 (.14)	0.007
Average highest PEEP^a^	6.2 (2.3)	5.4 (0.6)	0.09
Average lowest PEEP^a^	5.3 (1.6)	5.1 (0.3)	0.50
Average highest PIP^a^	19.3 (5.6)	14.5 (4.2)	0.001
Average lowest PIP^a^	16 (4.7)	13.3 (4.2)	0.02

All values are presented as percentages except ^a^in mean (standard deviation) and ^b^in median (25,75 quartiles). *CPAP* continuous positive airway pressure, *FiO2* fraction of inspired oxygen, *PEEP* positive end expiratory pressure, *PIP* peak inspiratory pressure, *aOR* adjust odds ratios, and *CI* confidence intervals, that were calculated in a regression model.

Table 4 provides more information on the characteristics of pneumothorax at different gestational age groups. Regardless of the GA category, most infants had chest radiographs in records before pneumothorax occurred. The use of chest tube and the need for mechanical ventilation after pneumothorax diagnosis was more frequently encountered in the lowest GA category. Only 3% of infants ≥ 35 weeks were intubated after the diagnosis of pneumothorax as 8% were intubated before pneumothorax that changed to 11% after pneumothorax. Intubation was required in 8% in the 29-34 weeks as it changed from 27% before pneumothorax to 35% after pneumothorax. Infants ≤28 weeks were mostly (77%) intubated before pneumothorax and that did not change after pneumothorax.

**Table 4:** Characteristics of pneumothorax in different gestational age groups.

	≥ 35 weeks	29-34 weeks	≤ 28 weeks
Laterality of pneumothorax			
Right	44	37	51
Left	23	39	33
Bilateral	33	24	16
Chest x-ray prior to pneumothorax	65	75	82
Support before pneumothorax			
None	18	8	0
Free flow oxygen	9	2	0
Bubble CPAP	17	6	3
Non-invasive mechanical ventilation	48	57	20
Intubation and mechanical ventilation	8	27	77
Chest tube placement	20	57	77
Support after pneumothorax			
None	30	8	3
Free flow oxygen	18	6	0
Bubble CPAP	5	0	3
Non-invasive mechanical ventilation	36	51	20
Intubation and mechanical ventilation	11	35	74
Age of pneumothorax			
Day 0	76	41	31
Day 1	7	24	8
Day 2	6	20	15
Day 3	3	8	5
Day 4	2	2	10
Day 5	2	4	5
Day 6	0	0	5
Day 7	2	2	3
Day 8 and beyond	2	0	18
Age of pneumothorax, days^a^	0.75 (1.7)	1.28 (1.6)	3.13 (3.0)

All values are presented as percentages except ^a^in mean (standard deviation).

Before the implementation of b-CPAP in the NICUs, the prevalence of pneumothorax in non-intubated infants was 0.35% compared to 0.41% after implementation, odds ratio 0.85 (95% CI: 0.62-1.16), *p* value 0.35.

## Discussion

The study reported an overall incidence of pneumothorax of 0.53%, with higher rates in preterm infants: 0.39% in those ≥35 weeks GA, 4.0% in 29–34 weeks GA, and 4.6% in ≤28 weeks GA. Across all GA groups, most pneumothoraces occurred within the first 24 hours of life, though the timing of diagnosis varied slightly in the most preterm infants. Several clinical variables were significantly associated with pneumothorax, with notable differences among GA categories. For neonates ≥35 weeks GA, pneumothorax was more common in male infants and was significantly associated with maternal chorioamnionitis, and delivery room CPAP. Among mechanically ventilated infants, exposure to higher PIP was a significant factor. In the 29–34 weeks GA cohort, SGA status, maternal diabetes, and surfactant administration were significantly associated with pneumothorax, along with exposure to higher FiO_2_ and PIP in ventilated infants. For infants ≤28 weeks GA, delivery room intubation and higher PIP exposure in the NICU emerged as key risk factors. The introduction of b-CPAP in the study NICUs during 2018–2019, did not change the prevalence of pneumothorax in non- intubated infants across any GA category. This suggests that while b-CPAP was widely adopted as a primary respiratory support modality, it may not have substantially influenced the overall pneumothorax risk.

The findings of this study aligning with previous studies demonstrating that pneumothorax is more frequent in preterm infants and those requiring advanced respiratory support. Male sex was associated with increased risk for pneumothorax in full term and late preterm and with GA ≥ 35 weeks. The association between male sex and increased pneumothorax risk in late preterm and term neonates is consistent with existing literature, which suggests that male neonates may have greater vulnerability [9–11]. This increased susceptibility could be explained by differences in lung development and surfactant composition between sexes. Male infants are known to have a lower lecithin/sphingomyelin ratio and reduced concentrations of saturated phosphatidylcholine in amniotic fluid, indicating less lung maturity [12].

The link between chorioamnionitis and pneumothorax supports previous evidence that intrauterine inflammation contributes to lung injury and altered pulmonary mechanics, predisposing affected infants to barotrauma. The association between chorioamnionitis and pneumothorax is to some extent controversial as a pervious report showed a decreased risk for pneumothorax with chorioamnionitis [13]. The relationship between antenatal steroid exposure and pneumothorax should be interpreted with caution. While prenatal steroids are expected to enhance lung maturity and reduce the risk of pneumothorax, the observed positive association in this study may reflect a confounding relationship. Steroid administration could serve as a surrogate marker for increased illness severity or anticipated respiratory compromise in high-risk infants, rather than a direct contributor to pneumothorax development.

The strong association between pneumothorax and increased PIP in mechanically ventilated infants underscores the importance of lung-protective ventilation strategies to mitigate barotrauma risk.

The study reported the novel finding that implementation of b-CPAP in the NICU did not associate with changes in pneumothorax incidences. The lack of significant change in pneumothorax rates pre- and post-b-CPAP introduction in the current study may reflect institutional practices emphasizing careful CPAP utilization with detailed and standardized guidelines, and careful use of b-CPAP pressure, with avoidance of using distending pressure higher than 5-6 cmH2O. B-CPAP is not necessarily like other types of CPAP giving he oscillatory nature of the pressure offered by b-CPAP. Therefore, the study offers safety data from 3 NICUs showing no pneumothoraces increases with the use of b-CPAP in term and preterm infants. A previous report associated the use of delivery room CPAP with pneumothorax [8]. The current study emphasizes the need for cautious use of CPAP in the delivery room, particularly in infants with additional risk factors for pneumothorax. While CPAP is a crucial intervention for preventing intubation and surfactant therapy, excessive pressures or inappropriate use may increase the risk of lung overdistension and air leak syndromes. The findings also emphasize the importance of optimizing mechanical ventilation settings to minimize barotrauma, particularly in preterm infants who require invasive respiratory support.

Pneumothorax is most seen immediately after birth, when higher distending pressures may be needed to ventilate the neonate, and in the resolution period of RDS, when pulmonary compliance may rise quickly [14]. In a single center study comparing term and preterm neonates with pneumothorax, most term neonates presented with symptoms within 24 hours after birth and recovered with a low frequency of invasive treatment [11]. The current study demonstrated that the timing of presentation was very early in infants ≥ 35 weeks GA, 76% happened in first day of life, in contrast only 31% happened in first day of life in infants ≤ 28 weeks GA. Although delivery room CPAP has been associated with an increased risk of pneumothorax,⁸ most infants in this study had at least one chest radiograph without pneumothorax before it was detected on subsequent imaging. This suggests a time lag between the initial respiratory management and the radiographic appearance of pneumothorax. While the underlying mechanism remains unclear, this novel observation highlights the need for further clinical and/or preclinical studies to elucidate the pathophysiological processes responsible for this delayed presentation.

The choice of treatment for pneumothorax depends on the severity of symptoms, varies from expectant management to invasive treatment with either thoracentesis and/or thoracostomy [15]. In this study, the clinical presentation of pneumothorax was usually benign in infants ≥ 35 weeks GA and mostly resolved spontaneously, eighty percent of those babies did not require chest tube placement compared to 33% of newborns ≤ 28 weeks GA. Preterm cases were also more likely to become intubated and receive surfactant.

A major strength of this study is the large, multi-NICU dataset spanning over seven years, allowing for a comprehensive analysis of pneumothorax incidence and risk factors across different GA groups. The use of a standardized data collection form and rigorous statistical methods, including logistic regression to adjust for confounding variables, enhances the validity of our findings. The comparison between b-CPAP and other sorts of non-invasive respiratory support is novel in this study. However, there are limitations to consider. As a retrospective study, the analysis is subject to potential documentation inconsistencies. The study did not assess other ventilatory components beyond PIP and PEEP, which could provide further insights into the role of specific respiratory support strategies in pneumothorax risk. Additionally, while we stratified subjects by GA, other potential confounders such as specific delivery room management were not fully accounted for. While controls were matched by gestational age and year of admission, potential residual confounders—such as differences in illness severity or delivery room management—may still influence the observed outcomes. Although the multivariable model included several clinical (e.g., maternal risk factors, Apgar scores, and delivery room intubation) and physiological indicators (e.g., PaO₂, PaCO₂, and oxygen requirement), these variables may not fully capture the complexity of illness severity in preterm infants. Furthermore, in certain subgroups, particularly among infants <28 weeks’ gestation, some adjusted odds ratios were associated with wide confidence intervals, limiting interpretability and reflecting reduced statistical power. These limitations should be considered when interpreting the findings.

This study provides insights into the epidemiology and risk factors for neonatal pneumothorax in a large, contemporary cohort. The findings highlight the role of perinatal factors, respiratory management strategies, and mechanical ventilation parameters in pneumothorax development. While b-CPAP implementation did not significantly alter pneumothorax incidence, ongoing efforts should focus on refining non-invasive ventilation strategies and optimizing respiratory support in at-risk infants. Future research should explore advanced lung-protective ventilation approaches in the prevention of pneumothorax. Further investigation into the impact of prenatal factors such as inflammation and fetal lung maturation on pneumothorax risk could also provide novel insights into prevention strategies.

In conclusion, neonatal pneumothorax is associated with the use of mechanical ventilation, intubation and higher PIP. Furthermore, the use of bubble CPAP was not associated with increased risk of pneumothorax, however, delivery room CPAP was associated with pneumothorax in newborns ≥ 35 weeks GA.

## Data Availability

The datasets generated and/or analyzed during the current study are available from the corresponding author on reasonable request.
